# Bibliographical

**Published:** 1893-01

**Authors:** 


### Bibliographical.

Methods of Filling Teeth. An Exposition of Practical Methods which will Enable the Student and Practitioner of Dentistry successfully to Prepare and Fill all Cavities in the Human Teeth. By Rodrigues Ottolengui, M.D.S. With 236 illustrations, giving the exact representations of all classes of cavities and their management.

This work consists in the main of bringing together in one volume the series of articles that have appeared in the *Dental Cosmos* during the last year or more.

The work deals almost exclusively with manipulation, and in this it is full and complete, dealing very clearly and concisely with almost every imaginable operation in the process of filling teeth, so that by studying this work closely and following the descriptions no one would be excusable for imperfect work.

The detail in filling teeth is more fully described and illustrated in this work than has ever before been attempted. No attempt has been made in the work to treat of the various diseases and pathological conditions to which the teeth are subject, nor even to deal with the varieties of teeth in their structural aspects--this perhaps is well.

The doctor has well illustrated the idea that doing one thing at a time secures the more perfect results. There are but few things, indeed, in the work that we would be disposed to criticise, and even these are not of special import. The work upon the whole is a very valuable one, and should be in the hands of not only the student, but of the general practitioner as well; and every description and illustration should be well studied; whether the reader would adopt them or not, no one can help being benefitted by a careful examination of the book.

The work is published by the S. S. White Dental Manufacturing Co., and in respect to its mechanical execution we need say nothing further than this. It can be obtained of any Dental depot.

---

THE RISE, FALL AND REVIVAL OF DENTAL PROSTHESIS. An introductory lecture by B. J. Cigrand, B.S., D.D.S., Professor of Dental Prosthesis in the American College of Dental Surgery.

This little work recently from the press is one of general interest to the profession. It contains more of the early history of dental prosthesis than will be found in any other single volume or place. It reaches back to the early period of Egyptian civilization, giving what is said and recorded concerning dentistry in these early periods. Hebrew dental art is mentioned, Chinese and Arabian also. Greek dental art forms one section, as does also Roman dental art. Many interesting and curious statements are made in regard to each of these countries on this subject. Etrurian dental art also comes in for its description. Cuts are given of specimens that were found in Etrurian tombs, dating back twenty-five hundred years, the illustrations are given of these. Illustrations are also given of the products of dental prosthesis of the present day, and we are able to compare them with the products of more than two thousand years ago.

The author discusses European dental art of the later times, giving many interesting things concerning its development and growth for the last two hundred years. One section is devoted to American dental art, in which its growth in this country has been very fully portrayed. The little book is certainly an interesting one, and is calculated to broaden the ideas of any one who is interested in such matters.

It may be had of Severinghaus & Beilfuss, printers and publishers, 448 Milwaukee Avenue, Chicago, or any Dental Depot. Price, $1.50.

